# Estimation of migraine prevalence considering active and inactive states across different age groups

**DOI:** 10.1186/s10194-023-01624-y

**Published:** 2023-07-11

**Authors:** Marco Piccininni, Ralph Brinks, Jessica L. Rohmann, Tobias Kurth

**Affiliations:** 1 Institute of Public Health, Charité – Universitätsmedizin Berlin, Charitéplatz 1, 10117 Berlin, Germany; 2 Center for Stroke Research Berlin, Charité – Universitätsmedizin Berlin, Charitéplatz 1, 10117 Berlin, Germany; 3 Chair for Medical Biometry and Epidemiology, Faculty of Health/School of Medicine, Witten/Herdecke University, Alfred-Herrhausen-Straße 45, 58455 Witten, Germany; 4 Max Delbrück Center for Molecular Medicine in the Helmholtz Association (MDC), Robert-Rössle-Straße 10, 13125 Berlin, Germany

**Keywords:** Migraine, Prevalence, Classification, Remission, Epidemiology, Population studies

## Abstract

**Background:**

Migraine is a very common headache disorder on the population level, characterized by symptomatic attacks (activity). For many people with migraine, the migraine symptoms intermittently or permanently cease during their lifetime (inactive migraine). The current diagnostic classification of migraine considers two states: active migraine (having migraine symptoms within the last year) and not having active migraine (including both individuals with inactive migraine and those who never had migraine). Defining a state of inactive migraine that has gone into remission may better capture the trajectories of migraine across the lifespan and contribute to a better understanding of its biological processes. We aimed to quantify the prevalence of never, active, and inactive migraine separately, using modern prevalence and incidence estimation methodology to better describe the complexity of migraine trajectories at the population level.

**Methods:**

Using a multistate modeling approach, data from the Global Burden of Disease (GBD) study, and results from a population-based study, we estimated the transition rates by which individuals moved between migraine disease states and estimated prevalences of never, active and inactive migraine. We used data from the GBD project and a hypothetical cohort of 100,000 people with a starting age of 30 and 30 years of follow-up, both in Germany and globally, stratified by sex.

**Results:**

In Germany, the estimated rate of transition from active to inactive migraine (remission rate) increased after the age of 22.5 in women and 27.5 in men. The pattern for men in Germany was similar to the one observed on the global level. The prevalence of inactive migraine among women reaches 25.7% in Germany and 16.5% globally at age 60. For men, the inactive migraine prevalence estimates at the same age were 10.4% in Germany and 7.1% globally.

**Conclusions:**

Considering an inactive migraine state explicitly reflects a different epidemiological picture of migraine across the lifecourse. We have demonstrated that many women of older ages may be in an inactive migraine state. Many pressing research questions can only be answered if population-based cohort studies collect information not only on active migraine but also on inactive migraine states.

**Supplementary Information:**

The online version contains supplementary material available at 10.1186/s10194-023-01624-y.

## Introduction

Migraine is a chronic-intermittent primary headache disorder and one of the most common adult pain disorders at the population level. The disease is treated in specialized headache centers by neurologists but also, in many cases, by general practitioners. Migraine is associated with a significant reduction in quality of life for those affected, an increased economic burden, and various comorbidities [1–4].

The diagnostic classification of migraine and other headache disorders is based on the International Classification of Headache Disorders (ICHD), currently in its 3rd edition [5]. While this diagnostic classification system for migraine works well in the clinical setting and has improved treatment decision-making [6], several issues arise when seeking to classify this disease status longitudinally. More specifically, the classification system relies on symptoms that are currently present or that have presented within the one-year period prior to assessment to classify the respective headache disorders [5]. On the other hand, the ICHD specifies that for genetic studies and some other uses, any occurrence during the entire lifetime is used [5]. Thus, the ICHD allows for either a definition of migraine that is purely based on active symptoms or a lifetime migraine definition (a prevalent “pool” that, once entered, can only be exited by dying), which ignores the distinction between individuals who have active and inactive migraine.

An explicit state of inactive migraine or recurrence pattern over time are features currently not incorporated into migraine classification. However, without an objective measurement of migraine status (e.g., biomarker), incorporating such states can be important. Explicitly classifying an inactive migraine state could contribute to the understanding of underlying biological mechanisms or prognosis prediction for people with migraine. Disease states have also been important for other diseases, such as multiple sclerosis, influencing prognosis and treatment choices [7, 8]. An inactive migraine state may also be useful in individual risk assessment, especially in light of known interrelationships with comorbid conditions [9, 10].

While some studies on the trajectory from childhood to adult migraine exist [11], limited data are available on migraine activity trajectories across the entire lifecourse at the population level, and most inferences come from prevalence studies [12–14]. These studies indicate that the prevalence of active symptomatic migraine reaches its peak around the age of 40 and then declines with increasing age in both men and women. This relationship is frequently illustrated in the typical migraine activity age-prevalence curve, characterized by a bell shape for both women and men [12, 13, 15]. Such an age-prevalence curve is also published in the Global Burden of Disease (GBD) study results, which relies on the ICHD classification [16].

The GBD approach is centered around understanding and quantifying disease burden, and as such, it focuses on direct, symptomatic aspects of migraine. Therefore, the GBD Study defines migraine disease as migraine activity. This is the same strategy used to define migraine prevalence in most of the prevalence studies found in the literature [14].

This strategy, while useful in evaluating disease burden, is incomplete and only yields a partial picture of the epidemiology of migraine. Therefore, in this work, we aimed to quantify the prevalence of active and inactive migraine separately using a modern prevalence and incidence estimation methodology to reflect the complexity of migraine trajectories at the population level [17, 18]. Specifically, we investigated migraine activity remission rates (transition from active to inactive migraine) across all ages and constructed age-prevalence curves of migraine separately for active and inactive migraine, both for Germany and on a global level.

## Methods

### Models definition

Our work relies on the use of multistate models [17–19]. These models are based on the definition of states, represented by nodes, and transition rates from one state to the other, represented by directed vertices between the nodes (usually represented as arrows).

**Fig. 1:**
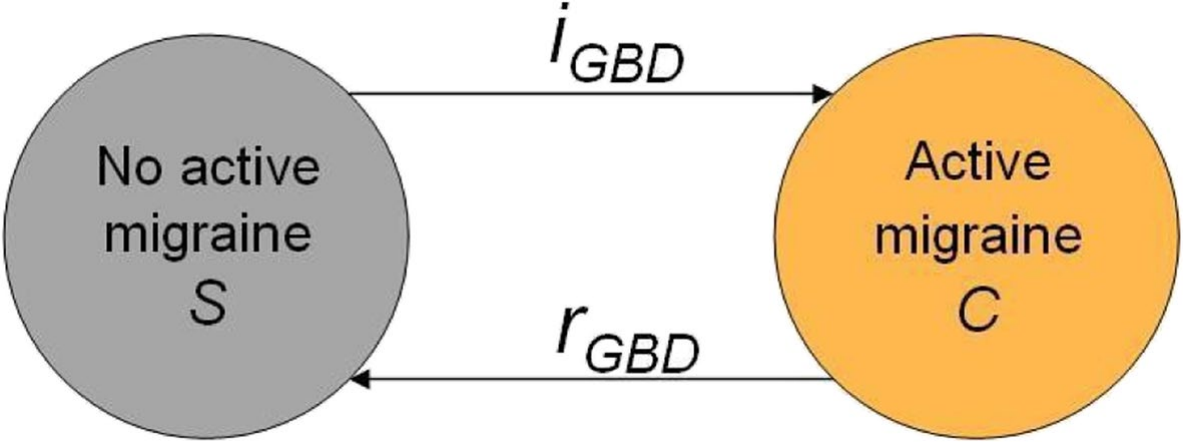
GBD migraine model. Global Burden of Disease data assumes the existence of only two relevant migraine states (no active migraine and active migraine). No differential mortality is assumed (therefore, no death state is depicted)

In our study, we specifically aimed to extend the GBD model to explicitly consider the state of inactive migraine, that is, migraine disease without symptomatic activity within the last year. Our interpretation of the GBD model is shown in Fig. 1. The GBD model assumes only two relevant health states for individuals with regard to migraine: (1) having no active migraine (S), which includes *both* individuals who never experienced migraine and those who previously had active migraine that has since gone into remission (inactive state), and (2) active migraine (C) defined as symptomatic migraine activity within the last year (in accordance with the ICHD criteria [5]). Since the GBD model assumes no differential mortality among those with active migraine versus no active migraine, the state of death was not included (Fig. 1).

**Fig. 2:**
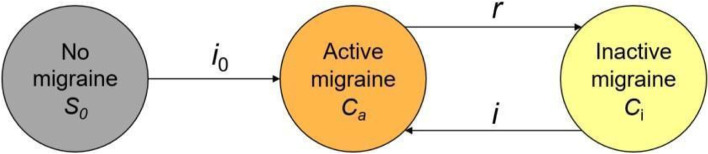
Extended model. In this model, the state of ‘No active migraine’ from the GBD model (Fig. 1) is split into two states: ‘No migraine’ and 'Inactive migraine’

To account for the state of inactive migraine, we split the state of “No active migraine” (S) presented in the GBD model (Fig. 1) into two distinct states: “No migraine” (S_0_) and “Inactive migraine” (C_i_). For clarity, we denote active migraine as (C_a_). Our Extended model is presented in Fig. 2. Our Extended model is mathematically related to the GBD model. We detail these relationships in Additional file 1: Appendix A.

### Estimation of the transition rate from active migraine to inactive migraine

We calculated the prevalence of active migraine as the proportion of individuals who had active migraine symptoms within the last year (compatible with the ICHD classification [5]). Similarly, we defined the prevalence of inactive migraine as the proportion of individuals who did not experience migraine activity in the last year but had previously been in the active migraine state. We do not refer to lifetime prevalence unless otherwise specified.

To estimate the prevalence of inactive migraine, we needed to estimate the transition rate *r* in Fig. 2, which represents the remission rate of active migraine (rate of transition from active to inactive migraine) and mathematically corresponds to the *r*_GBD_ rate from the GBD model (Additional file 1: Appendix A).

Using the GBD Results Tool [20], we extracted values for the incidence rate and prevalence of active migraine, along with their uncertainty intervals (UI), for all possible combinations of the following variables: year (1990 to 2019), sex (male, female), location (Germany or Global), and age group (< 1 year, 1 to 4, 5 to 9, 10 to 14, …, 90 to 94, and 95 plus). We additionally created a continuous variable for age, corresponding to the midpoint of the age groups and equal to 97.5 for the highest age category. Separately by sex and location, we built models to predict the prevalence of active migraine *p* (which is equivalent to *p*_*a*_; Additional file 1: Appendix A) and incidence *i*_GBD_ based on age and year. We then used a differential equation describing the relationship between remission rate, prevalence of active migraine, and incidence of active migraine to estimate the remission rates *r*_*GBD*_*.* In the equation, we relied on the two models to estimate the values of *p* and *i*_*GBD*_ by age and year, for each sex and location combination. The 95% confidence intervals (CIs) and the point estimate of the remission rates were obtained by resampling the original GBD data. Details about the modeling of *p* and *i*_GBD_, the estimation of *r*_*GBD,*_ and its confidence interval are provided in Additional file 1: Appendix A.

### Integration of additional, external information to quantify the rates

To identify all the quantities needed to fully specify the Extended model, the information provided by the GBD is not sufficient. We therefore relied on the information reported in Rasmussen et al. about the lifetime prevalence of migraine by age and sex [21]. We chose this study because it used a randomly selected population sample and a standardized assessment tool, reported lifetime prevalences by age groups and sex, and involved a population very similar to the German one (Denmark) [21]. Having information about the lifetime prevalence of migraine allowed us to obtain both the incidence of active migraine among those who never had migraine (*i*_0_) and the prevalence of never having had migraine (*p*_0_) from a single study. To achieve this objective, we exploited some known mathematical relationships between these quantities, detailed in Additional file 1: Appendix A.

From Fig. 2 of Rasmussen et al. [21], we obtained values of migraine lifetime prevalence (and corresponding 95% confidence intervals) for the age-groups 25–34, 35–44, 45–54, and 55–64 for women and men, separately. We attributed the value of the lifetime prevalence to the midpoint age in each age group. To account for variability for each age-sex group, we sampled 5,000-lifetime prevalence values. The values were sampled from a normal distribution with the mean equal to the lifetime prevalence reported from the 1991 Rasmussen et al. study for each age-sex group and with the standard deviation equal to half of the distance between the reported upper and lower 95% confidence limits divided by 1.96. Negative lifetime prevalence values were treated as missing values.

A linear regression model with the logit of the lifetime prevalence as the dependent variable was run for each of the 5,000 sets of lifetime prevalence values. The linear regression included age and sex as predictors and an interaction term between age and sex. Out of the 5,000 linear regressions, the ones with coefficients leading to a decreasing age-dependency (in males or females) were considered not admissible and were therefore excluded (see Eq. (3) in Additional file 1: Appendix A), leaving us with 1,830 valid sets of coefficients.

We then used the median values of the coefficients (b_0_, b_1_, b_2_, and b_3_) across all valid sets to estimate the value of *i*_0_(*a*) and *p*_0_(*a*) for males and females. The value of *i*_0_(*a*) was obtained by plugging the median regression coefficients into Eq. (5) from Additional file 1: Appendix A, while the value of *p*_0_(*a*) was obtained as one minus the prediction of *q*_0_(*a*), obtained using the median regression coefficients b_0_, b_1_, b_2_, and b_3_.

### Estimation of inactive migraine prevalence

To run the Extended model (Fig. 2), we set up a discrete Markov model, in which the transitions between the states are approximated by finite time steps. The details of the Markov model and the estimation procedure for all rates are described in Additional file 1: Appendix A. Briefly, we simulated a cohort of 100,000 individuals with a starting age of 30 for each combination of location (Germany, Global) and sex (male, female). Next, we simulated the start of the observation of each cohort in 1990, and we fixed the follow-up length to 30 years. After initializing the values for the number of individuals in each state (never migraine S_0_, active migraine C_a_, inactive migraine C_i_) at the beginning of the follow-up to the prespecified initial conditions, we applied the recursion formulas at every step (see Additional file 1: Appendix A). We chose a step length of 0.01, and at every step, we obtained the number of individuals with active migraine, inactive migraine, and never migraine, relying on the estimated age- and sex-specific transition rates for the chronological year and the location of interest. Finally, we estimated the age-specific prevalence for each sex and location combination as the proportion of individuals in the simulated cohorts who were in a particular state at the specific step during the follow-up.

As a sensitivity analysis, we repeated the estimation of inactive migraine prevalence relying on external information provided by the more recent study from Le et al. [22] (instead of Rasmussen et al. [21]). Details of the sensitivity analysis are reported in Additional file 1: Appendix A.

All analyses were conducted in R (version 4.2.0) and RStudio (version 2022.02.2 + 485).

### Patient and public involvement

For our project, we did not explicitly involve patient groups. However, multiple authors suffer from migraine headaches. Our study has implications for the public awareness of migraine across the lifecourse and has been stimulated by many discussions with experts and people who have migraine.

### Availability of data and materials

We used publicly available data from the GBD project [20] as well as estimates from two publications [21, 22].

### Ethics approval

As our study estimated quantities by using publicly available data from the GBD project and from two published population-based studies [21, 22], ethics approval was not required.

## Results

**Fig. 3:**
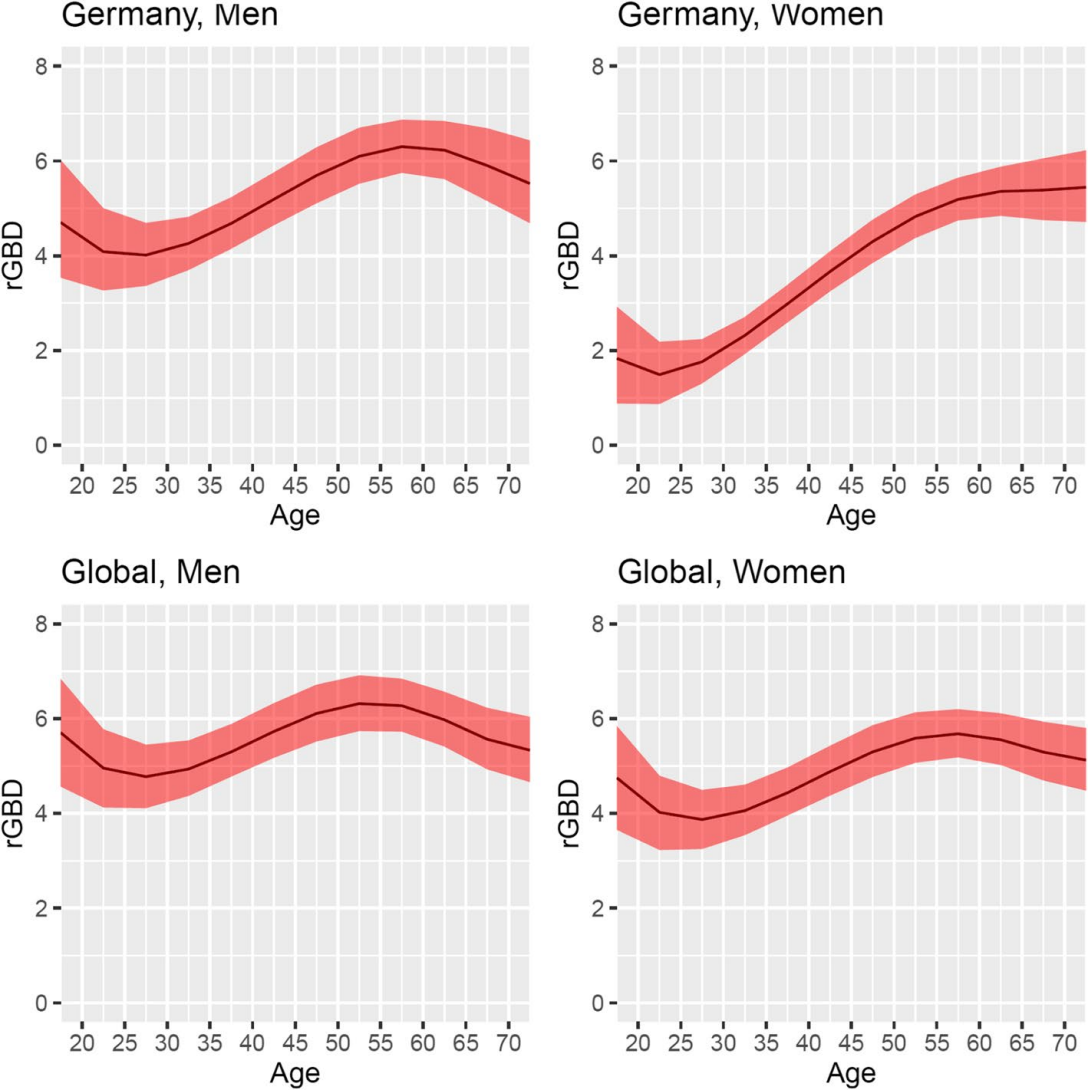
Remission (transition from active to inactive migraine) rates per 100 person-years at risk, estimated from the GBD data for 2019 by sex, location, and age (age range: 17.5 to 72.5). The 95% confidence interval (red area) was obtained by resampling

The estimated migraine activity remission rates (along with 95% confidence intervals) across the full age range from 17.5 to 72.5 years by sex and location (global, Germany) for the year 2019 are reported in Fig. 3. For Germany, we estimated the remission rates at age 17.5 to be 4.70 (3.54 to 6.02) per 100 person-years for men and 1.83 (0.88 to 2.92) per 100 person-years for women. After a slight decline, the estimated remission rates for both sex groups start increasing (starting at age 27.5 in men and 22.5 in women). Remission rates among German men reach their peak (6.30, 5.75 to 6.87 per 100 person-years) at the age of 57.5, while the remission rate for German women does not start decreasing before the age of 72.5, reaching the value of 5.45 (4.71 to 6.22) per 100 person-years at this age.

The pattern observed for both men and women at the global level is similar to the one described for German men (Fig. 3).

**Fig. 4:**
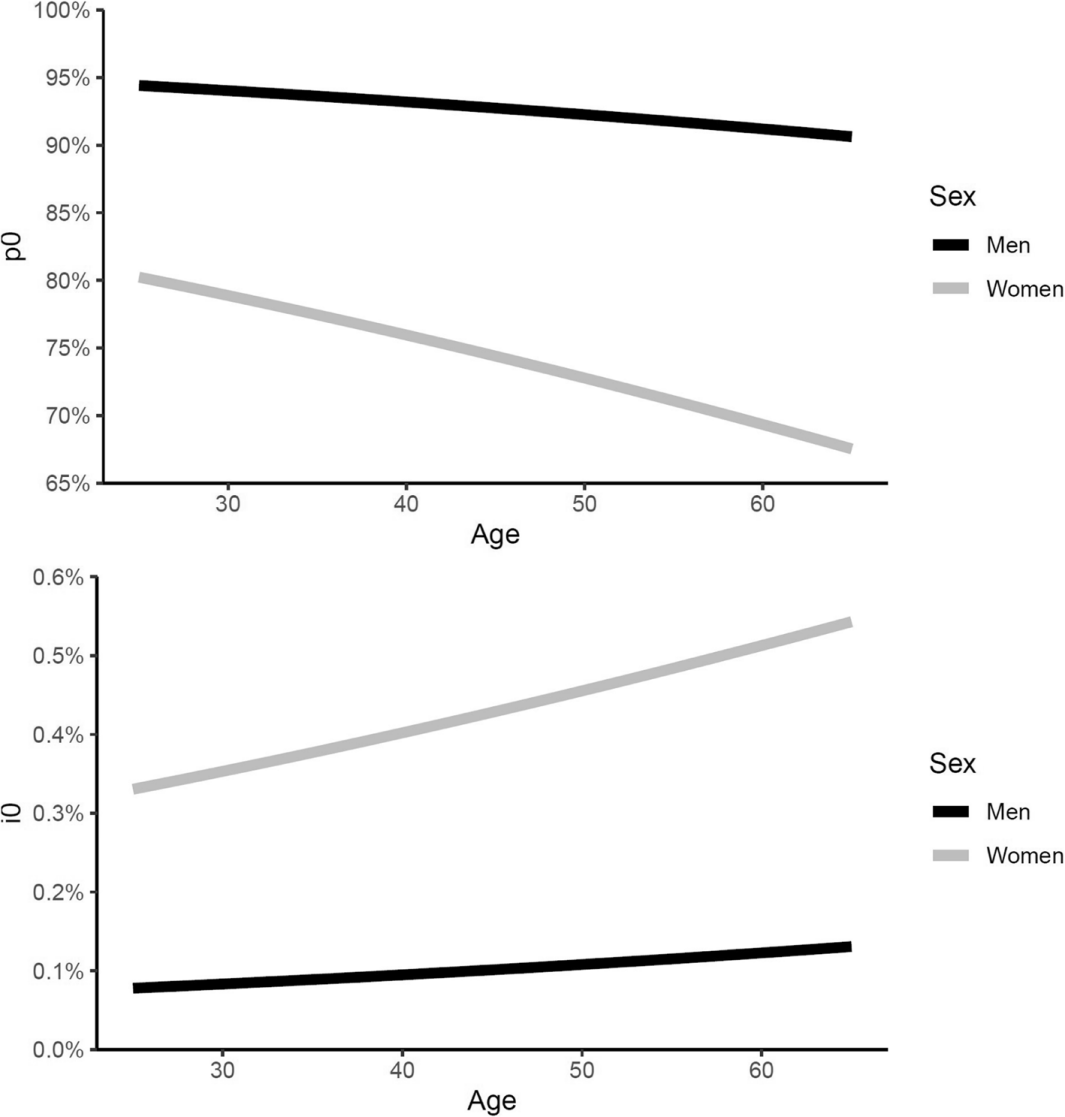
Estimated prevalence of never having had migraine (p_0_) (upper plot) and the incidence of active migraine within the last year among those who never had migraine (i_0_) (lower plot) by age and sex. The estimates were obtained by modeling the lifetime prevalence estimates from Rasmussen et al. [21]

In Fig. 4, we show the estimated prevalence of never having had migraine (*p*_*0*_) and the incidence of active migraine among those individuals who never had migraine (*i*_*0*_) by age and sex. As expected, there is a steeper decline in the prevalence of never having had migraine, which, in women, drops from 80.2% at age 25 to 67.5% at age 65 (and from 94.4% to 90.6% in men). On the other hand, *i*_*0*_ was found to increase across ages for both men and women, from 0.08% in men and 0.33% in women at age 25 to 0.13% in men and 0.54% in women at age 65.

**Fig. 5:**
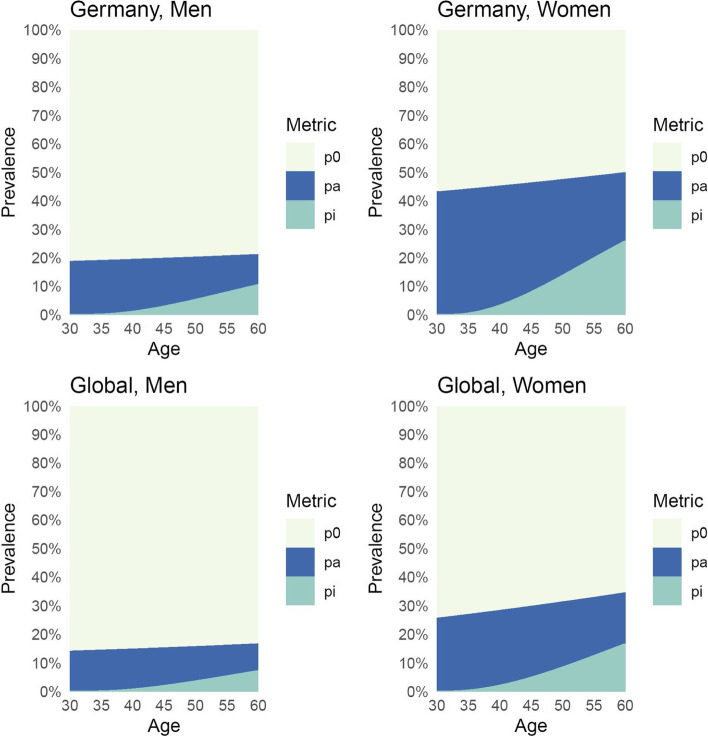
Prevalence of individuals who never had migraine (*p*_*0*_, in light yellow), with active migraine within the last year (p_a_, in blue), and inactive migraine (*p*_*i*_, in green), estimated from the Extended model (Fig. 2). Prevalences were estimated from a theoretical cohort of 100,000 individuals with a starting age of 30 years old and a follow-up of 30 years for each combination of location (Germany, Global) and sex (men, women). We simulated the start of the observation of the cohorts in the year 1990

The results of the Markov model’s simulated cohorts are presented in Fig. 5. The prevalence of inactive migraine (after having had migraine) both at the German and Global level is lower for men compared to women, as expected. The prevalence of inactive migraine is nearly zero at age 30 and increases steadily for all combinations of sex and location. According to our model, the prevalence of inactive migraine among women reaches 25.7% in Germany and 16.5% globally at the age of 60. This means that 25.7% of all 60-year-old women living in Germany do not have migraine symptoms but have previously experienced migraine at some point in their lives. Among men, the prevalence of inactive migraine reaches 10.4% in Germany and 7.1% globally at the age of 60. The sensitivity analysis using more recent data yielded similar results (see Online Additional file 1: Fig. 1).

## Discussion

In this study, we quantified age-specific prevalence estimates, of (1) individuals never having migraine, (2) individuals having active migraine, and (3) individuals having inactive migraine stratified by sex, in both Germany and on a global level using GBD data. We further quantified the remission rates of migraine activity for both Germany and globally. This was accomplished using theoretical cohorts with simulated follow-up based on available data from the GBD and observed values obtained from a large, population-based study. As expected, remission states are particularly relevant for women; based on our estimates, in women aged 60 years, approximately 17% globally and 26% in Germany can be classified as having an inactive migraine state.

While it is known that migraine is more prevalent in women than men and the prevalence of active migraine, after reaching its peak, generally decreases in older ages, with a less pronounced decline observed in men compared to women [15, 23], separate prevalence estimates for active and inactive migraine have not, to the best of our knowledge, previously been reported in the scientific literature. Notably, this operationalization goes beyond the current ICHD system, which does not make a category of inactive migraine explicit [5].

Little is known about the biological processes resulting in the different activity states of migraine and what consequences for other organ systems, such as the vascular system, occur. There is, for example, evidence that women with an inactive migraine state may have a more unfavorable vascular risk profile as measured by the Framingham risk score for coronary heart disease [9]. Other work has suggested that a history of migraine (compared with no migraine history) is associated with an increased likelihood of depression [10]. Continued research to address these and other pressing questions accurately can only be conducted if population-based cohort studies collect information not only on active migraine or lifetime history of migraine but also on inactive migraine states.

### Further considerations on migraine states

Since migraine classification relies on migraine symptoms, the two non-migraine states we present, never migraine and inactive migraine, are both characterized by the absence of symptoms. According to our model, the inactive migraine state can only be reached after an individual has been in the active migraine state. We acknowledge that this may be an oversimplification of the underlying biological processes. For example, it may be possible that a subclinical (inactive) migraine state is present before any migraine symptoms appear.

Furthermore, it is important to consider diagnostic uncertainty, specifically, whether probable active migraine and definite active migraine represent different expressions of the same underlying migraine state or represent distinct states. Since 2017, GBD modeling accounts for both probable and definite defined migraine [24, 25]. However, Rasmussen et al. [21] (from which we used information for our hypothetical cohorts, predated the introduction of the probable migraine definition. This aspect may have introduced some inconsistency in the prevalence estimates and should generally be considered when comparing our estimates to other studies. It has been shown that changes in the definition of migraine can impact the prevalence estimates in population-based studies [23]. Importantly, we assumed that individuals with inactive migraine can only exit the inactive migraine prevalence pool through active migraine or death. However, it could be theoretically possible to transition back to a “pre-migraine” state, although currently, biological knowledge is lacking. Further work could explore how the inactive migraine state relates to the interictal state (between migraine attacks) of people with active migraine [26]. Lastly, excluding inactive or remitted migraine from current cross-sectional prevalence estimates may conceal the associated burden if a history of migraine confers disadvantage through missed opportunities that may persist post-remission (i.e., in individuals who no longer have migraine if the disease is defined exclusively by its active state). This particular component of migraine-attributable burden, which may be substantial for some individuals, may be important to consider in future studies.

### Strengths and limitations

Strengths of our study include the use of a modern methodological approach to estimate prevalences. This allowed for estimation of the remission rates and the prevalence of inactive migraine across age groups and sex both in Germany and globally.

Several limitations should be considered when interpreting our results. First, the use of our migraine lifetime prevalence estimates by age group and sex from a study from Denmark that included only people aged 25–64 [21] may be viewed as a limitation. However, as this population-based study used standardized methods and reported the necessary data for our estimates, it allowed us to estimate two needed quantities consistently using a single study.

Moreover, in our sensitivity analysis using more recent data about lifetime migraine prevalence from Denmark [22], we obtained similar results in terms of inactive migraine prevalence. Second, our modeling approach assumed that the lifetime prevalence of migraine is stable across chronological years and transportable to Germany and the global level. Since there is an indication that active migraine prevalence may have increased in the years since the study data were collected [14], this would mean that our estimates underestimate the true prevalence of active migraine. Lastly, as with all model-based studies, our approach relies on multiple assumptions, approximations, and choices when faced with contradicting results, which we have reported transparently. One of the main assumptions we relied on was the absence of differential mortality between the different states. This assumption simplifies the estimation problem, and we determined it to be reasonable since it is also an assumption used by the GBD to generate its estimates [16]. However, while migraine does not increase all-cause mortality in women [27, 28], there is some evidence of increased risk of cardiovascular-specific mortality for women with migraine with aura [28], and one population-based study from Iceland suggested an increased risk of all-cause mortality in men with migraine [29]. Though it would add considerable complexity, future work could explore the potential implications of accommodating the increased mortality risk in some population subgroups.

### Implications and further research

While many details about the underlying pathological processes of migraine are being uncovered [4], identifying migraine by overt, observable symptoms alone may result in missing an important part of the full picture of migraine and its potential consequences both on the individual and population level.

For this reason, we believe it is crucial to collect information over time on (1) symptomatic, active migraine, (2) inactive migraine (remission of migraine symptom activity among individuals who had migraine in the past, ideally supplemented with information on when a person’s migraine symptoms stopped) and (3) resumed migraine activity after a period of inactivity (and ideally, how long the inactive state persisted). Only once such information becomes available in longitudinal studies can migraine trajectories be adequately studied at the population level.

Although in clinical practice, it is well established that migraine activity fluctuates [30], a more formal consideration of a migraine inactivity state may help to improve the management of migraine. Not formally classifying nor collecting data about a remission state has important ramifications for migraine research. Trajectories of active and inactive states of disease can provide important information about biological aspects and may have important consequences for treatment management as well as prognosis, especially in light of specific comorbidities. For example, clinical course descriptions/phenotypes (consisting of active/non-active states both with and without progression) in multiple sclerosis have resulted in distinct disease classifications that not only are useful in prognosis [7, 8] but also have been linked to specific inherited different genetic patterns [31] and can help guide treatment management strategies.

In summary, considering an inactive migraine state explicitly reflects a different epidemiological picture of migraine across the life course. We have demonstrated that many women of older ages may be in a migraine inactivity state. With a continuously aging population, this represents a substantial number of individuals. Therefore, more research on inactive migraine is needed to generate insights about this large population stratum, especially if the inactive state of migraine puts individuals at an increased risk for other diseases. Explicitly considering an inactive migraine state will facilitate further investigation into whether such patients should be assessed differently in the clinical setting.

## Supplementary Information

**Additional file 1:** **Appendix A.** Methods details and sensitivity analysis results.

## Data Availability

We used publicly available data from the GBD project [20] as well as estimates from two publications [21, 22].
