# Supplementary material for: Estimation of migraine prevalence considering active and inactive states across different age groups
Source: J Headache Pain. 2023 Jul 11;24(1):83. doi: 10.1186/s10194-023-01624-y (PMC10334692; doi:10.1186/s10194-023-01624-y)
Supplement: Supplementary file 1 — Additional file 1: Appendix A. Methods details and sensitivity analysis results. [file 10194_2023_1624_MOESM1_ESM.docx]

# Appendix A. Methods details and sensitivity analysis results

**Mathematical relationship between the GBD model and our Extended model**

Let *p*_0_, *p*_a_ and *p*_i_ be the respective proportions of the population in states S_0_, C_a_ and C_i_, then it holds that *p*_0_ + *p*_a_ + *p*_i_ = 1.

First, we note that the prevalence *p* of active migraine in Figure 1 corresponds to *p*_a_ in Figure 2. Therefore, *p*_a_ = *p*. Moreover, by definition, since the rates *r*_GBD_ and *r* refer to the same type of events and the same population at risk, we find a further equivalence between the two models:

*r*_GBD_ = *r* (1)

Another relationship between the rates in the GBD model (Figure 1) and the Extended model (Figure 2) is:

*i_GBD_* ᐧ (*p*_0_ + *p*_i_) = *i*_0_ *p*_0_ + *i p*_i_ (2)

This follows intuitively from the fact that the number of individuals entering the pool of individuals with active migraine in the Extended model and the GBD model must be the same, even if in the GBD model, the individuals can only enter from one state (S), and in the Extended model, they can enter from two states (S_0_ and C_i_). A formal proof of the equivalence (2) can be found below.

*Proof of Equation (2).* To see why Eq (2) holds true, we notice that the "No active migraine" state in Figure 1 represents the pooled state of the two states "No migraine" and "Inactive migraine" in Figure 2. The rates *i*_0_ and *i* refer to the populations at risk *N p*_0_ and *N p*_i_, respectively, where *N* is the overall population size. Hence, we have *i*_0_ ~ #(transitions from *S*_0_ to *C*_a_)/(*N p*_0_) and *i* ~ #(transitions from *C*_i_ to *C*_a_)/(*N p*_i_). The notation '# 'means "number of" and '~' means "during a small time interval, with a length approaching zero".

As *i*_GBD_ ~ #(transitions into *C*)/[*N* ᐧ (1 - *p*)], we have *i*_0_ *p*_0_ + *i p*_i_ ~ [#(transitions from *S*_0_ to *C*_a_) +

#(transitions from *C*_i_ to *C*_a_)]/*N* = #(transitions from anywhere to *C*_a_)/*N* ~ *i*_GBD_ ᐧ (1 – *p*). From 1 – *p*

= 1 – *p*_a_ = *p*_0_ + *p*_i_, we obtain Eq (2).

# Details about the modeling of *p* and *i_GBD_*

The prevalence was obtained as a convex combination of the constant 0 and the prediction of a polynomial model fit on the GBD data. In the polynomial model, the prevalence was modeled as a sextic function (polynomial of sixth order) of age and interaction terms between the chronological year and each polynomial term of the sextic function. The weight of the convex combination was fixed to 1 before the age of 5. For ages above 5, the weight was fixed to the reciprocal of the age minus 5. This mathematical strategy was introduced to force the prevalence to be equal to zero before the age of 5, an assumption of the GBD data, as well as to avoid unnatural jump discontinuity in the function. The incidence was obtained using the same method, but the weight of the convex combination at age above 5 was fixed to be the reciprocal of the age minus 5, raised to the power of five. Goodness of fit of the prevalence and incidence prediction methods were graphically evaluated using the GBD data. The described procedure was repeated separately for each sex and location combination.

# Estimation of the transition rate *r_GBD_*

Under the assumption of no excess mortality, remission rates by age and year were estimated using the following formula:

*r*_GBD_(*t,a*) = ( (1 – *p*(*t,a*)) ᐧ *i*_GBD_(*t,a*) – 𝜕*p*(*t,a*) ) ᐧ *p*(*t,a*)^-1^

Which derives from Equation 3 in Brinks et al. [[1]](https://paperpile.com/c/ALZISn/51LXW) when no differential mortality is present. Where 𝜕*p*(*t,a*), the partial derivative, was approximated by:

𝜕*p*(*t,a*) = ( *p*(t + 0.1, *a* + 0.1) – *p*(t - 0.1, *a* - 0.1) ) ᐧ (2 ᐧ 0.1)^-1^

The functions *p*() and *i*_GBD_() were approximated using the prediction methods described in the section “Details about the modeling of *p* and *i_GBD_”*. The migraine remission rates for prevalence estimates lower than zero were assumed to be zero. In order to produce 95% confidence intervals (CI) for the remission rate estimates, we repeated the procedure described above and computed the remission rates from 5,000 resamplings of the original GBD prevalence and incidence data. In each resampling, variability was induced by drawing the value of the prevalence (or incidence) for an age-year group from a normal distribution with mean equal to the GBD-reported prevalence (or incidence) value and standard deviation equal to half of the difference between the upper and the lower UI limits divided by 1.96. The 2.5%, 97.5%, and 50% percentiles of the remission rate estimate distribution for each age and year were used as lower limits of the CI, upper limits of the CI, and point estimates, respectively. The described procedure was repeated separately for each sex and location combination.

**Justification for the integration of additional, external information to quantify all rates** The lifetime prevalence *q*_0_ is equal to the proportion of individuals who experienced migraine thus far in their lifespan at a given moment. Therefore, *q*_0_ represents the proportion of individuals who, at a given moment, are not in the state S_0_. From this follows the equality:

*q*_0_ = 1 – *p*_0_.


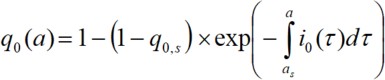
Moreover, lifetime prevalence at a specific age *a, q*_0_(*a*), and ever-migraine incidence *i*_0_*(a*) are mathematically related as follows:

*(3)*

where *q*_0,s_ is the lifetime prevalence at the start *a*_s_ of the first age group. The integral in Equation

(3) is usually called the “cumulative incidence”. The intuition behind this relationship is that the proportion of individuals *not* in the state S_0_ at a given age, ( *q*_0_(*a*) ), is purely the consequence of how many individuals were not in the state S_0_ at the first available value of age (*a*_s_) and with which intensity the individuals left the state S_0_ (*i*_0_) for all the years from *a_s_* to *a*. A more formal proof is reported below. From Eq (3), we see that the lifetime prevalence *q*_0_ is always a

non-decreasing function of age *a*. Given the no excess mortality assumption (also made by the GBD), any decreasing function is mathematically not possible (not admissible).

*Proof of Equation (3)*. With the assumption of no excess mortality due to the ever-migraine state, the relation between *q*_0_ and *i*_0_ is given by the ordinary differential equation:

𝜕*q*_0_/𝜕*a* = (1 – *q*_0_) *i*_0_. (4)

Integrating the linear ordinary differential equation (4) and using the initial condition *q*_0_(*a*_s_) = *q*_0,s_

yields Eq (3).

It has been proven useful to model prevalences as the inverse of logit, i.e., expit-functions, which are defined by expit(*x*) = exp(*x*){exp(*x*) + 1}^-1^. The derivative of expit(*x*) has a specific form:

expit*'* (*x*) = expit(*x*) {1 – expit(*x*)}.

Therefore, if we can model the proportion *q*_0_(*a*) as the expit of an appropriate known function of age, *q_0_*(*a*) = expit( *f_0_*(*a*) ), we know immediately the derivative of *q*_0_(*a*):

𝜕*q_0_*/𝜕*a* = *q_0_'*(*a*) = *f_0_'*(*a*) *q_0_*(*a*) {1 – *q_0_*(*a*)}

Combining this result with the known result presented in Equation (4), we obtain that:

𝜕*q*_0_/𝜕*a* = *f_0_'*(*a*) *q_0_*(*a*) ᐧ {1 – *q_0_*(*a*)} = (1 – *q*_0_(*a*)) *i*_0_(*a*) It follows immediately that *i*_0_(*a*) = *f_0_'*(*a*) *q_0_*(*a*) = *f_0_'*(*a*) ᐧ expit( *f_0_*(*a*))

Let us model the logit of the prevalence *q*_0_(*a*) as *f*_0_(*a*) = b_0_ + b_1_ *a* + b_2_ *s* + b_3_ *s* ᐧ *a*

with *a* being age in years and *s* encoding sex (0 = “female”, 1 = “male”). If this model provides a reasonable fit to *q*_0_, we obtain:

*i*_0_(*a*) = (b_1_ + b_3_ *s* ) ᐧ expit(b_0_ + b_1_ *a* + b_2_ *s* + b_3_ *s* ᐧ *a*) (5)

# Setup of the Markov model

We set up a discrete Markov model, in which the transitions between the states in the Extended model (see Figure 2) are approximated by finite time steps (with length *dt*). For a birth cohort of size N aged *a*_0_ in year *t*_0_, we applied the following recursion formulas:

(*t*_k+1_, *a*_k+1_) = (*t*_k_ + *dt*, *a*_k_ + *dt*)

S_0_(*t*_k+1_, *a*_k+1_) = S_0_(*t*_k_, *a*_k_) ᐧ {1 – [*i*_0_(*t*_k_, *a*_k_) + *m*(*t*_k_, *a*_k_)] ᐧ *dt*}

C_a_(*t*_k+1_, *a*_k+1_) = C_a_(*t*_k_, *a*_k_) ᐧ {1 – [*r*(*t*_k_, *a*_k_) + *m*(*t*_k_, *a*_k_)] ᐧ *dt*} + S_0_(*t*_k_, *a*_k_) ᐧ *i*_0_(*t*_k_, *a*_k_) ᐧ *dt* + C_i_(*t*_k_, *a*_k_) ᐧ *i*(*t*_k_, *a*_k_) ᐧ

*dt*

C_i_(*t*_k+1_, *a*_k+1_) = C_i_(*t*_k_, *a*_k_) ᐧ {1 – [*i*(*t*_k_, *a*_k_) + *m*(*t*_k_, *a*_k_)] ᐧ *dt*} + C_a_(*t*_k_, *a*_k_) ᐧ *r*(*t*_k_, *a*_k_) ᐧ *dt*

and the initial conditions:

S_0_(*t*_0_, *a*_0_) = N *p*_0_ (*t*_0_, *a*_0_)

C_a_(*t*_0_, *a*_0_) = N *p*_a_ (*t*_0_, *a*_0_) C_i_(*t*_0_, *a*_0_) = N *p*_i_ (*t*_0_, *a*_0_)

If *p*_0_(*t*_0_, *a*_0_) was found to be higher than 1 – *p*_a_ (*t*_0_, *a*_0_), the latter was used as the initial value of

*p*_0_(*t*_0_, *a*_0_).

In the previous sections, we explained in detail how we identified the functions *r*(*t*_k_, *a*_k_), *i*_0_(*t*_k_, *a*_k_), and *p*_0_(*t*_0_, *a*_0_). In order to estimate the remission rates, we also had to approximate *p*(*t,a*) and

*i*_GBD_(*t,a*). As we noted above, *p*_a_(*t*_0_, *a*_0_) and *p*(*t,a*) are the same quantity. Therefore, we obtained the predictions for *p*_a_(*t*_0_, *a*_0_) from the same prediction model we used to approximate *p*(*t,a*).

Having *p*_0_(*t*_0_, *a*_0_) and *p*_a_(*t*_0_, *a*_0_), we obtained *p*_i_(*t*_0_, *a*_0_) by arithmetic difference, since we know that these three prevalence rates must sum to 1. The incidence *i*(*t*_k_, *a*_k_) was then obtained by solving Equation (2). Finally, the mortality *m*(*t*_k_, *a*_k_) was obtained by modeling the all-cause mortality estimates, as extracted from the GBD Results Tool [[2]](https://paperpile.com/c/ALZISn/spBOG), by age and year for each combination of sex and location. We chose to model mortality with a linear regression built in the same way as the aforementioned polynomial regression used for modeling prevalence and incidence.

# Sensitivity analysis

The sensitivity analysis consisted of re-running the main analysis as previously described aside from replacing the procedure to obtain b_0_, b_1_, b_2_, and b_3_ described in the section “Integration of additional, external information to quantify the rates” with the following elements:

We extracted data from Figure 3 in Le et al. [[3]](https://paperpile.com/c/ALZISn/BSi3b) about the 2002 lifetime prevalence of migraine by sex and age in Denmark, using the online application WebPlotDigitizer [[4]](https://paperpile.com/c/ALZISn/PvsoJ). We then fitted a linear regression model with the logit of the lifetime prevalence as the dependent variable. As in the main analysis, the linear regression included age and sex as predictors and an interaction term between age and sex. The intercept and coefficients obtained by this model represented the b_0_, b_1_, b_2_, and b_3_ that were, in turn, used to estimate *i*_0_(*a*) and *q*_0_(*a*). The results of the Sensitivity analysis are illustrated in **Online Figure 1**.


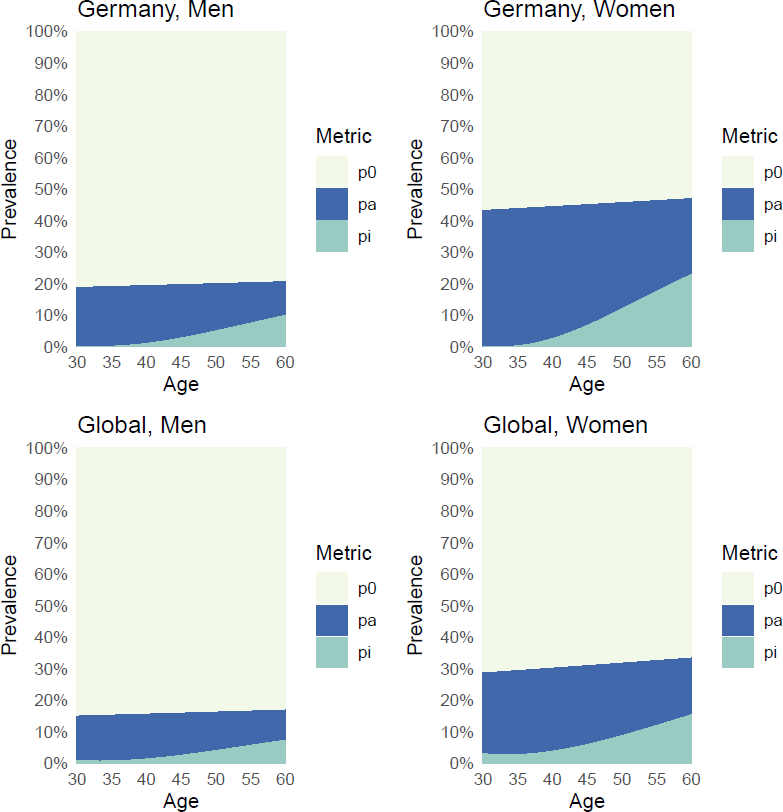


**Online Figure 1. Prevalence of individuals who never had migraine (*p_0_*, in light yellow), with active migraine (p_a_, in blue), and with inactive migraine (*p_i_*, in green), estimated from the sensitivity analysis.** Prevalences were estimated from a theoretical cohort of 100,000 individuals with a starting age of 30 years and 30 years of follow-up for each combination of location (Germany, Global) and sex (men, women). We simulated the start of the observation of the cohorts in 1990. The sensitivity analysis differs from the main analysis since external

information from Le et al. [[3]](https://paperpile.com/c/ALZISn/BSi3b) (instead of Rasmussen et al. [[5]](https://paperpile.com/c/ALZISn/Ipsxn)) was used.

# References

1. [Brinks R, Landwehr S (2015) Change rates and prevalence of a dichotomous variable:](http://paperpile.com/b/ALZISn/51LXW) [simulations and applications. PLoS One 10:e0118955](http://paperpile.com/b/ALZISn/51LXW)
2. [GBD Results Tool.](http://paperpile.com/b/ALZISn/spBOG) [http://ghdx.healthdata.org/gbd-results-tool.](http://ghdx.healthdata.org/gbd-results-tool) [Accessed 23 Mar 2021](http://paperpile.com/b/ALZISn/spBOG)
3. [Le H, Tfelt-Hansen P, Skytthe A, et al (2012) Increase in self-reported migraine prevalence in](http://paperpile.com/b/ALZISn/BSi3b) [the Danish adult population: a prospective longitudinal population-based study. BMJ Open 2.:](http://paperpile.com/b/ALZISn/BSi3b) [https://doi.org/](http://paperpile.com/b/ALZISn/BSi3b)[10.1136/bmjopen-2012-000962](http://dx.doi.org/10.1136/bmjopen-2012-000962)
4. [Rohatgi A WebPlotDigitizer - Extract data from plots, images, and maps.](http://paperpile.com/b/ALZISn/PvsoJ) [https://automeris.io/WebPlotDigitizer/.](https://automeris.io/WebPlotDigitizer/) [Accessed 18 Oct 2022](http://paperpile.com/b/ALZISn/PvsoJ)
5. [Rasmussen BK, Jensen R, Schroll M, Olesen J (1991) Epidemiology of headache in a](http://paperpile.com/b/ALZISn/Ipsxn) [general population—A prevalence study. J Clin Epidemiol 44:1147–1157](http://paperpile.com/b/ALZISn/Ipsxn)
